# Cancer-related anxiety, COVID-19, and the oncologist: the formation of a ‘Balint’ process group

**DOI:** 10.46439/psychiatry.1.004

**Published:** 2021

**Authors:** Daniel C. McFarland

**Affiliations:** Department of Medicine, Lenox Hill Hospital, Northwell Health, New York, NY, USA

## Introduction:

### The Dynamic of Cancer-Related Anxiety among Patients and Vicarious Distress of Clinicians

The management of anxiety and distress in patients with cancer is stressful for the oncology clinicians who treat them [1]. Unfortunately, psychosocial care for patients with cancer is not universally available or standardized [2]. Referrals from oncology services to psychological serves are often not initiated early enough, may not be encouraged from medicine or surgical services, and are subsequently foregone or patients do not follow up beyond a single appointment [3]. As purveyors of cancer-related information, oncologists often find themselves in situations where their patients are reluctant to engage psychosocial care for various reasons (e.g., stigma, additional appointments, or just prioritizing oncology care] but remain highly symptomatic, which can be disruptive to their quality of life, their families, and following through with their oncology care [4,5]. The consequence is detrimental to their overall quality of life and cancer related mortality and places undue stresses on primary oncology services who are not equipped to manage complicated psychological stressors of their patients [6]. Inadvertently, distress is easily transmitted to cancer care teams and oncologists who are caring for them [7,8].

### The Interface of COVID-19 and Psychosocial Stressors in Oncology Care

In addition to the usual stresses of having cancer, the COVID-19 pandemic has altered the usual delivery of cancer care making every encounter with a medical facility fraught with danger, increasing the sense of isolation and loneliness that patients feel during this pandemic [9,10]. This is particularly devasting for patients experiencing hospitalization, side effects from cancer treatment, or end of life care [11]. The direct effect of COVID-19 on the psychological health of patients with cancer is understandably problematic and runs parallel to the anxiety experienced by oncology clinicians who fear contagion or may be experiencing burnout or a sense of moral distress by witnessing injustices in care delivery or vicarious traumatization [11]. The psychological burden experienced by healthcare systems across the world is unprecedented in the modern era [12]. The need to protect patients obtaining cancer care from COVID-19 infections led to drastic changes in the delivery of healthcare including changes in work schedules and roles, the rapid implementation of telehealth capabilities and practices, procedural delays, treatment alterations, delays in ambulatory visits, changes in end of life care (isolation] and suspension of clinical trials, and implementation of policies on the usage of personal protective equipment (PPE] [13]. Some practices converted 50% of visits to a virtual, distanced platform [14]. Oncology patients who become infected with COVID-19 have worse outcomes than patients without cancer [15].

### The Role of Consult-Liaison Psychiatry

Consult-liaison psychiatry tends to focus more on psychiatric consultations than on liaison activities [16]. Historically, the liaison role of CL-psychiatry was impactful and helped establish institutional medical cultures and practices [17]. Similarly, the sub-field of psycho-oncology relied on liaison work when oncology practice was more centralized in the hospital [18]. Co-localization is an ever-present issue due to busy schedules and emergencies. As a result of the pandemic, however, virtual presence is more frequently accepted and even expected, perhaps as a surrogate for co-localization.

The stresses of oncology practice and the management of cancer-related distress and anxiety may be co-managed to some extent by the liaison activity of conducting in-person or virtual Balint groups, which may be led by CL psychiatrists or other trained mental health professionals with specific Balint group training [19].

### The Role of Liaison ‘Balint’ Groups

The ‘Balint’ group was created in the 1950 by the English psychoanalysis Enid Balint and is used around the world to help other physicians outside of mental health with the stresses of difficult patients and/or medical situations [20]. The guiding therapeutic principle of the Balint group is perspective-seeking, reestablishing alliance, replenishing the doctor-patient relationship, reestablishing alliance, and enhancing the wellbeing of the physician by increasing knowledge of skill around the doctor-patient relationship [21]. In essence, it is an experiential education that stands to benefit physicians who may be struggling with inter-relationship issues with their patients or self-care. Medical education along with postgraduate training places greater emphasis on acquisition of medical science and the ability to incorporate medical science into medical practice. This is a laudable goal especially with the ever-increasing amount of medical information that needs to be assimilated into practice. However, patients tend to rate communication as the highest priority for their care along with competence since they are necessarily associated [22]. This mismatch can place considerably strain on the doctor-patient relationship as communication models have shifted to shared-decision making [23–25].

Incorporating ‘Balint’ group training and experiences is consistent with the tenants of patient-centered medicine [26]. Evidence has been collected for its feasibility, especially within primary care disciplines, but is incomplete regarding its effectiveness across various disciplines of medicine [27]. The added convenience of a virtual presence may make their use even more appealing in our current state of medical practices [28]. Group activity confers therapeutic benefit for physicians who may feel isolated in practice and or struggling with ongoing burnout. While this would not be a therapeutic vehicle for more serious mental health issues that have been reported among physicians, it has prevention potential and is appropriate for adaptation to physician wellness.

### Tapping the Potential of ‘Balint’ Groups for Oncology and Palliative Care Physicians

Oncologists and other cancer clinicians outside of mental-health have limited training on interpersonal communication, doctor-patient relationship, transference/countertransference and limited supervision on effectively dealing with difficult or personality disordered patients. In general, patients who are reluctant to receive psychosocial services use their primary medical provider as a surrogate support for mental healthcare [29,30]. This is mostly seen as acceptable given the positive transference that develops toward the oncologist, which can be leveraged to enhance support and provide direction in terms of disease expectations and emotional coping. The patient-oncologist alliance has been found to lower suicidal ideation [31]. The provision of psychosocial support often originates from oncology clinics. In fact, the biggest predictor of mental healthcare follow-up is the oncologist attitude towards the mental health issue, which may also be compromised by increased moral distress and/or more distanced care due to the COVID-19 pandemic [32].

Alongside a global pandemic is the ongoing epidemic of physician distress and burnout [1]. While there is evidence of staff distress due to the pandemic, a newly found flexibility has also emerged in the form of willingness to use electronic platforms to connect with colleagues and patients [33]. There is also increased attention and prioritization of clinician welling [14]. Individual clinicians and organizations are seeking interventions to address the compounded stresses experienced by clinicians today.

‘Balint’ groups for oncology clinicians who are in training or experienced have demonstrated feasibility and draw upon common principles that enhance the doctor-patient relationship and the wellbeing of clinicians [34]. The traditional ‘Balint’ group may be adapted and improved using virtual platforms and the newly found motivation to improve physician wellbeing during this global pandemic.
